# Imaging of aqueous outflow in health and glaucoma. Justifying the re-direction of aqueous

**DOI:** 10.1038/s41433-024-02968-8

**Published:** 2024-03-01

**Authors:** Jed A. Lusthaus

**Affiliations:** 1 Department of Ophthalmology, Sydney Eye Hospital, Sydney, NSW Australia; 2 Discipline of Ophthalmology, The University of Sydney, Sydney, NSW Australia

**Keywords:** Glaucoma, Outcomes research

## Abstract

A wave of less invasive surgical options that target or bypass the conventional aqueous outflow system has been incorporated into routine clinical practice to mitigate surgical risks associated with traditional glaucoma drainage surgery. A blanket surgical approach for open-angle glaucoma is unlikely to achieve the desired IOP reduction in an efficient or economical way. Developing a precise approach to selecting the most appropriate surgical tool for each patient is dependent upon understanding the complexities of the aqueous outflow system and how devices influence aqueous drainage. However, homoeostatic control of aqueous outflow in health and glaucoma remains poorly understood. Emerging imaging techniques have provided an opportunity to study aqueous outflow responses non-invasively in clinic settings. Haemoglobin Video Imaging (HVI) studies have demonstrated different patterns of aqueous outflow within the episcleral venous system in normal and glaucomatous eyes, as well as perioperatively after trabecular bypass surgery. Explanations for aqueous outflow patterns remain speculative until direct correlation with findings from Schlemm’s canal and the trabecular meshwork are possible. The redirection of aqueous via targeted stent placement may only be justifiable once the role of the aqueous outflow system in IOP homoeostasis has been defined.

## Abstract (zh)

为了降低青光眼传统引流术相关的手术风险, 针对或绕过传统房水流出系统的微创手术方案已纳入常规临床实践。一刀切的开角型青光眼手术方法的有效性或达到经济性降眼压的预期效果。为每位患者选择最适合的术式取决于对房水引流系统的复杂性以及设备如何影响房水引流的了解。然而, 人们对健康眼和青光眼患者的房水平衡控制仍然知之甚少。新的眼科成像技术为在临床环境中以非侵入性方式研究房水流出反应提供了机会。血红蛋白视频成像 (HVI) 研究表明, 正常眼和青光眼以及小梁旁路手术后的围手术期, 巩膜外静脉系统中的房水引流模式各不相同。在与Schlemm管和小梁网的研究结果直接相关之前, 对房水引流模式的解释仍是推测性的。只有在明确了房水引流在眼压平衡中的作用后, 通过有针对性的支架置入重新引导房水的流出可能较为合理。

## Introduction

**Fig. 1:**
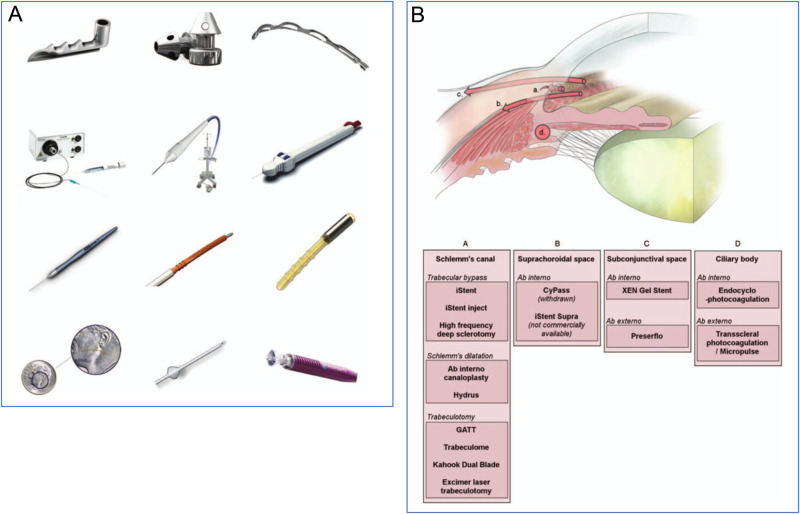
Minimally invasive glaucoma surgery (MIGS). **A** Examples of surgical devices used to reduce intraocular pressure. From top left to bottom right: iStent, iStent inject, Hydrus Microstent, iTrack, trabectome, TRAB 360, Kahook Dual Blade, CyPass Micro-stent (withdrawn from market), iStent Supra (not commercially available), XEN 45, PreserFlo Microshunt, MicroPulse G6 cyclophotocoagulation. **B** Diagrammatic representation of anatomical approaches to MIGS (GATT indicates gonioscopy-assisted transluminal trabeculotomy). Adapted diagrams reprinted from Gillmann K et al. [2] with permission from Wolters Kluwer Health, Inc.

Reducing intraocular pressure (IOP) to slow glaucomatous optic neuropathy remains the goal of all glaucoma surgery [1]. The introduction of multiple surgical devices to minimise the invasiveness and unpredictability of traditional glaucoma surgery has led to a shift in surgical decision-making. Minimally invasive glaucoma surgery (MIGS) provides options to improve aqueous drainage via the conventional (trabecular) outflow pathway, the supraciliary space or via the subconjunctival space (bleb-forming) with less tissue manipulation and a faster recovery (Fig. 1) [2–4]. However, despite favourable safety profiles, results have still been difficult to predict. Subconjunctival devices are hindered by scar tissue formation in a similar fashion to traditional trabeculectomy surgery. Use of the supraciliary space to lower IOP was halted in 2018 following the removal of the Cypass Microstent from the market due to concerns about corneal endothelial cell loss [5]. Other supraciliary drainage devices that minimally impact on endothelial cell health are becoming available [6, 7].

Devices that target the trabecular outflow system, in particular the iStent (Glaukos Corporation, USA), were the first MIGS devices used in clinical practice [8]. Safety profiles of trabecular bypass surgery (TBS) devices have been excellent [9–11]. However, unlike failure due to localised tissue scarring in subconjunctival and supraciliary approaches, there is no definitive explanation for variable success rates with TBS [12]. IOP reduction after TBS is known to be limited by episcleral venous pressure, but pathological factors within Schlemm’s canal [13, 14] and downstream in the episcleral venous system [15, 16] likely contribute to variable IOP reduction. MIGS devices that reduce trabecular resistance permit opportunistic study of the conventional AO system. This review will concentrate on Haemoglobin Video Imaging (HVI) studies in which the conventional aqueous outflow system was manipulated to illustrate different flow responses seen in health and glaucoma.

## A shift in glaucoma management

The traditional approach to IOP control in glaucoma involves a stepwise progression from topical drop therapy to selective laser trabeculoplasty (SLT) and finally glaucoma drainage surgery. The use of SLT has recently been proven to be an acceptable first-line treatment option [17]. Surgical lowering of IOP with MIGS devices has also shifted to earlier in the treatment paradigm [18]. Traditional drainage surgeries, trabeculectomy and tube shunt insertion, are associated with more effective IOP control, but also higher risks of infection, hypotony and loss of vision. However, longer-term clinical and cost-effectiveness data are required to enable accurate comparison with MIGS devices [19].

## Trabecular bypass surgery

The majority of physiological aqueous drainage occurs via the conventional (trabecular) outflow pathway [20]. TBS delivers aqueous more readily into Schlemm’s canal by reducing trabecular resistance. Despite successful device implantation 20–25% of cases do not achieve ≥ 20% unmedicated IOP reduction [21, 22]. Surgical success after TBS retrospectively implicates trabecular block as a significant contributor to IOP dysregulation. By deduction, failure of TBS to reduce IOP and improve AO indicates other potential mechanisms, which may include pathological changes within Schlemm’s canal (loss of elasticity or valvular disruption) or the episcleral venous system (raised episcleral venous pressure or vascular damage). Renewed interest in AO imaging has been generated by variable MIGS results, whilst also providing opportunities to refine concepts of glaucoma pathophysiology.

## Aqueous outflow imaging

Aqueous veins were first described by Ascher in 1942 with the use of slit lamp biomicroscopy [23]. Subsequently, the anatomical structure of the AO system has been documented in detail using ex vivo studies [24–26]. After its secretion by the ciliary body epithelium, aqueous is known to flow into the anterior chamber and drain through the trabecular meshwork into Schlemm’s canal. The distal AO system sequentially drains aqueous into collector channels, aqueous veins and episcleral veins before distributing their contents into the superior and inferior ophthalmic veins [20, 27]. Functional AO describes how aqueous flows through the system. Opportunistic AO imaging during intraocular surgery has been described using a number of techniques incorporating dyes [28–30]. However, intraoperative studies of AO are not physiological due to anaesthesia, pupil dilation, speculum use and perfusion of the anterior chamber, which all confound results [31].

Non-invasive imaging of the proximal AO system with phase-sensitive OCT has demonstrated dynamic movement of the trabecular meshwork in association with ocular pulsation [32, 33]. However, this is an indirect technique detailing movements of the surrounding structures rather than aqueous itself. AO imaging techniques have been hampered by the opaque sclera, which causes light scattering. Conversely, HVI exploits scleral reflectivity using a bandpass filter (540–580 nm) to create contrast between dark erythrocytes and clear aqueous [31]. HVI was initially developed to examine limbal microcirculations [34], with aqueous visualisation later being recognised as another application [35, 36]. Using HVI, aqueous drainage within the episcleral venous system can be identified non-invasively in a clinic setting without dye or contrast.

**Fig. 2:**
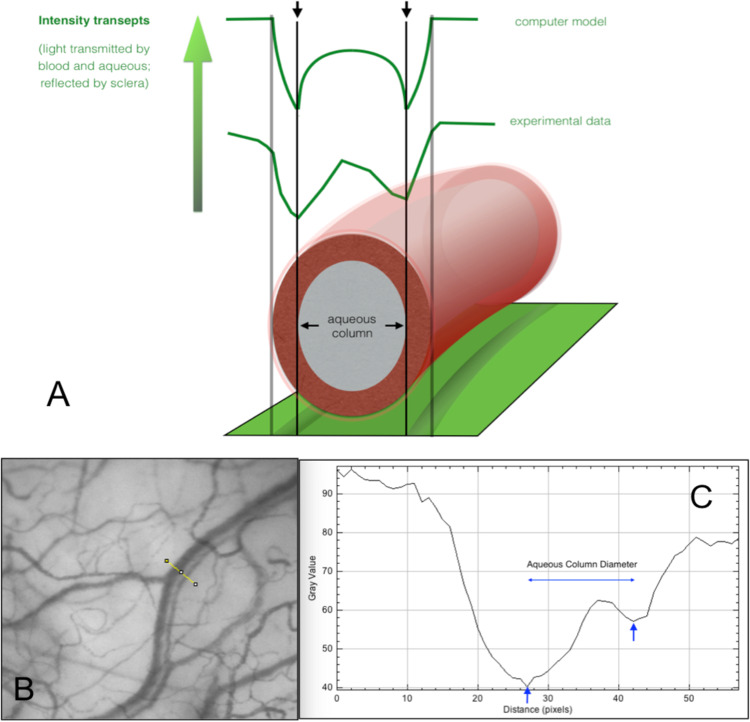
The method used to calculate aqueous column cross-sectional area. **A** Diagrammatic representation of aqueous column measurement from light intensity transepts. **B**, **C** An aqueous vein is pictured with a linear transept cutting across the vessel. The transept and corresponding graph are generated by Image J software. The aqueous column diameter equals the distance between the troughs (vertical blue arrows). The diameter in pixels is converted to aqueous column cross-sectional area (AqCA) in micrometres squared. Reproduced from Lusthaus et al. [31].

**Fig. 3:**
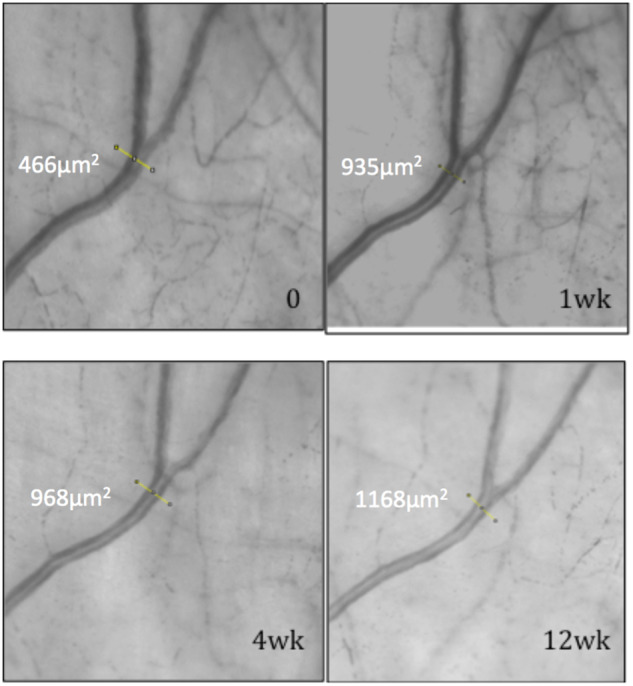
Improvement of aqueous outflow following trabecular bypass surgery as evidenced by gradual aqueous column cross-section area (AqCA) increase during the first 3 months after surgery. Linear transept is the site where AqCA measurement was taken. 0 = Preoperative laminar flow. AqCA increases 1 week after trabecular bypass surgery and this is maintained after 4 and 12 weeks. Adapted image reprinted from Lusthaus JA et al. [15] with permission from Wolters Kluwer Health, Inc.

Aqueous column cross sectional area (AqCA) in micrometres squared (μm^2^) is a surrogate measure for regional aqueous outflow that can be quantified using Image J open source software (Fig. 2) [15, 36]. A transept is generated within Image J at a nominated site along an aqueous vein. The same location can be measured longitudinally over time to assess the response to an intervention (Fig. 3) [15, 16, 37].

## Aqueous outflow in health and glaucoma

### Trabecular bypass surgery with iStent Inject

**Fig. 4:**
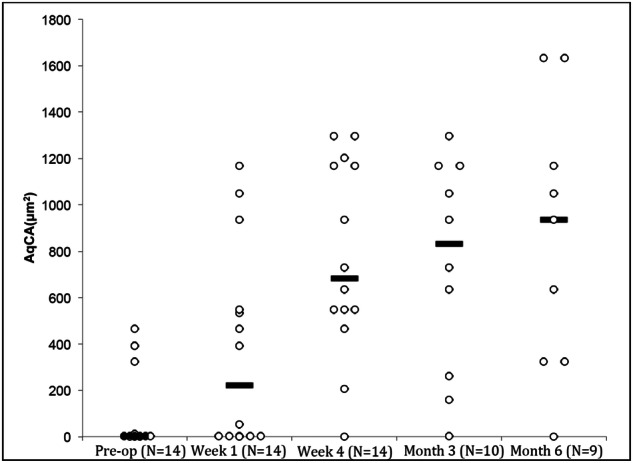
Gradual improvement in aqueous column cross-sectional area (AqCA) following trabecular bypass surgery after 4 weeks (*N* = 14; *P* = 0.002), 3 months (*N* = 10; *P* < 0.05) and 6 months (*N* = 9; *P* < 0.05). Black lines represent median AqCA. Figure reprinted from Lusthaus JA et al. [15] with permission from Wolters Kluwer Health, Inc.

Unpredictable TBS results have generated interest in finding methods to identify pre-operative predictors of surgical success. The emergence of HVI provided a new technique to study the effects of TBS on aqueous outflow in the hope of identifying such signs, however the complexity of the episcleral venous was quickly appreciated. The initial study examined 14 glaucomatous eyes for up to 6 months after iStent *Inject* insertion [15]. A gradual increase in median AqCA was seen, however there was a large variation between the peri-operative aqueous column sizes amongst the participants (Fig. 4) [15]. In a subsequent study examining IOP spikes within one month of iStent *Inject*, 20 glaucomatous eyes were imaged peri-operatively using HVI [16]. A group of 13 eyes had very low or no detectable AqCA at baseline. There was a significant increase of AqCA 4 weeks after surgery, likely implicating trabecular block as the primary mechanism of glaucoma in that group. The remaining 7 eyes had the largest pre-operative AqCA measurements, followed by a reduction after iStent *Inject* insertion in 6 eyes. This latter group is of particular interest because alleviation of trabecular block induced a decline in localised AO. Possible explanations include pathological changes within Schlemm’s canal or the episcleral venous system, localised obstruction to collector channels by the stent, or diversion of aqueous to another sector of the eye.

**Fig. 5:**
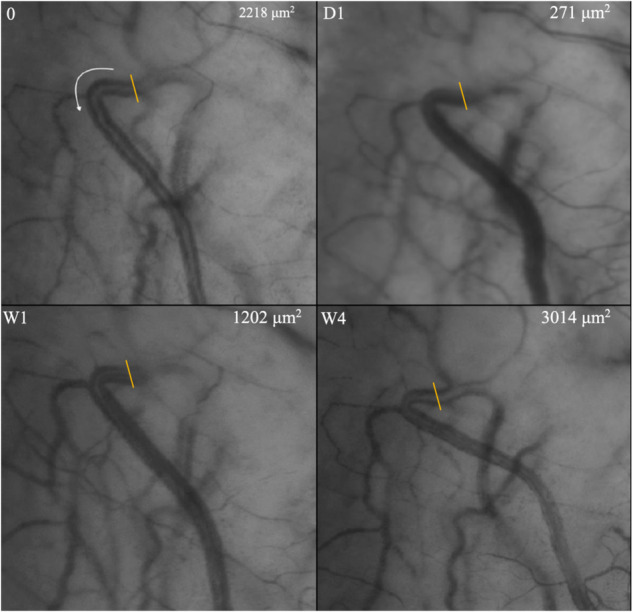
Aqueous outflow after standalone TBS seems to reduce 1 day after surgery (D1), begins to recover after 1 week when a 38% IOP spike developed (W1), and then improves after 4 weeks (W4). The white arrow in the preoperative image (**0**) represents the direction of aqueous drainage away from the limbus. AqCA reduces after surgery and then exceeds the preoperative measurement after 4 weeks. AqCA indicates aqueous column cross-sectional area, IOP intraocular pressure, TBS trabecular bypass surgery. Figure reprinted from Lusthaus JA et al. [16] with permission from Wolters Kluwer Health, Inc.

Qualitative analysis of post-operative aqueous drainage identified three patterns [16]. The most common finding, seen in 10 eyes, was recovery of aqueous laminar flow after 1 week, followed by improvement of flow after 4 weeks (Fig. 5). This pattern of improvement was associated with successful IOP reduction in almost all cases and was thought to represent surgical success. It is evident that a sudden reduction of trabecular outflow resistance does not lead to an immediate improvement in aqueous drainage. Gradual recovery of AO likely indicates an internal process within the eye designed to re-establish the equilibrium between aqueous production and drainage.

Secondly, in 6 eyes, aqueous laminar flow worsened or was lost at post-operative weeks 1 and 4. Two of these eyes demonstrated complete loss of the aqueous column with reversal of blood flow towards the limbus. In both cases this finding occurred despite an adjacently implanted stent. It is not yet possible to determine whether the stent induced flow reversal or regional pathological changes within Schlemm’s canal, or the episcleral venous system, prevented acceptance of aqueous. The latter hypothesis is supported by genome-wide association studies that suggest structures distal to the trabecular meshwork may be responsible for AO resistance in a proportion of patients [38]. Genetic studies indicate Schlemm’s canal is a lymphatic-like vessel and dysregulation of lymphangiogenesis may contribute to glaucoma pathogenesis [39]. This suggests a possible explanation for impaired aqueous outflow after trabecular-targeted treatments in some eyes.

Lastly, redirection of aqueous into neighbouring vessels was seen in 4 eyes. It is not known whether diversion of flow occurred due to preferential drainage induced by the stent or localised obstruction within Schlemm’s canal due to blood clotting or inflammation [16]. Aqueous outflow patterns after iStent *Inject* in glaucomatous eyes represent a range of pathophysiological responses to reduced trabecular resistance, however some of the changes may be related to the physical presence of the stent. Pre-operative AO characteristics to predict surgical outcomes are even less apparent. Identifying pathological manifestations of glaucoma within the episcleral venous system relies upon a clear definition of normal AO. However, the dynamic nature of the AO system makes it challenging to define. Manipulations of IOP within a clinic setting induce physiological responses that permit comparison between normal and glaucomatous eyes.

### The water drinking test

**Fig. 6:**
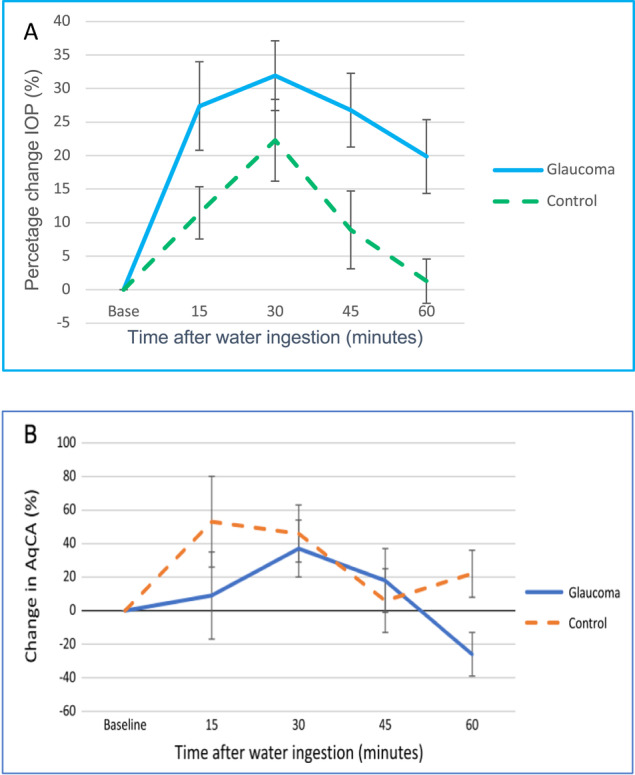
A Mean percentage change in intraocular pressure (IOP) during the water drinking test. Peak IOP was seen 30 min after water ingestion in both groups. **A** Mean percentage change in intraocular pressure (IOP) during the water drinking test. Peak IOP was seen 30 min after water ingestion in both groups. **B** The median percentage change in aqueous column cross-sectional area (AqCA) was compared at every interval. A poorly sustained AqCA response was seen in glaucomatous eyes with AqCA falling below baseline levels at 60 min. Adapted figures reprinted from Lusthaus JA et al. [37].

The water drinking test is commonly used to induce peak diurnal IOP [40–42] and was used to study AO responses in 20 glaucomatous eyes and 10 normal control eyes [37]. All participants consumed 10 ml of water per kilogram of body weight within 5 min. IOP and HVI were recorded every 15 min to complete a total of 60 min. Peak IOP of both groups was achieved after 30 min. In the glaucoma group, IOP remained elevated at the end of the study, but fell back to baseline in the control group (Fig. 6A). This was explained by the AqCA response, which increased in both groups, but was not sustained in the glaucoma group, falling below baseline by the end of the study (Fig. 6B). Impaired trabecular meshwork function may contribute to the drop off in AqCA in the context of elevated IOP. Collapse of Schlemm’s canal or raised EVP are other possible causes [37].

**Fig. 7:**
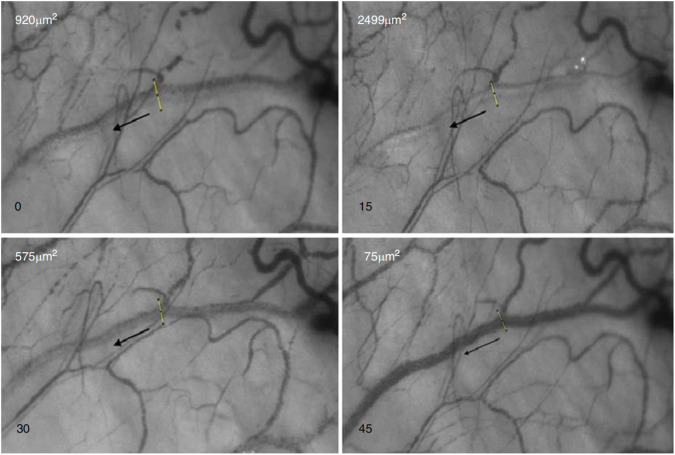
Aqueous column widening occurs within an episcleral vein of a glaucomatous eye 15 min after water ingestion. The aqueous column then reduces in size, almost disappears at 45 min and is not able to be detected after 60 min (not pictured). The site of aqueous column cross-section measurement is represented by a linear transept and the black arrows indicate direction of aqueous flow. Figure reprinted from Lusthaus JA et al. [37] with permission from Wolters Kluwer Health, Inc.

Three qualitative patterns of AO were induced by water ingestion [37]. Laminar flow with widening of the aqueous column, pulsatile displacement of blood (with and without flow reversal) and diffuse dilution of an episcleral vein. Widening of the aqueous column is most likely associated with a normal physiological response and persisted to the end of the study, predominantly in normal control eyes. In some glaucomatous eyes widening of the aqueous column terminated quickly (Fig. 7). Pulsatile flow reversal was seen in 5 glaucomatous eyes. Temporary restoration of stable aqueous laminar flow occurred in all 5 cases, but was not sustained [37]. Mixing of blood and aqueous occurs when the aqueous velocity is insufficient to produce an aqueous column. Instead, aqueous dilutes the blood column and AqCA cannot be measured. Both pulsatile flow reversal and diffuse dilution of an episcleral vein may indicate obstruction to aqueous drainage.

The identification of distinct aqueous outflow patterns, such as those described above, may provide a clinical adjunct to assist in the diagnosis and staging of glaucoma. It is not yet possible to predict surgical outcomes based on pre-operative AO characteristics. This is compounded by unpredictable redirection of aqueous drainage within Schlemm’s canal and the episcleral venous system following TBS. Defining perioperative aqueous flow dynamics may inform optimal stent placement or whether to bypass the system altogether with a subconjunctival or supraciliary approach. Further study of a larger cohort and corroboration with other TBS devices is required. There are also challenges to overcome before HVI can be considered as a clinical imaging tool.

## Challenges of aqueous outflow imaging

### Diurnal variation

Continuous buffering of IOP, controlled by structures within the aqueous outflow system, occurs in response to natural diurnal variations [13, 43]. However, the dynamic and variable nature of the AO system and IOP control leads to significant challenges during clinical study. Isolated IOP measurements during office hours have been shown to poorly reflect diurnal IOP control [44, 45]. Peak IOP most commonly occurs overnight [46–48]. IOP is also known to be affected by body position [46, 47, 49], fluid consumption [40–42, 50, 51], eye movement, eyelid blink, heart rate, breathing [52, 53] and some dietary factors (e.g. caffeine) [54, 55]. It follows that diurnal variation of AO occurs, however this has historically been harder to study. Aqueous humour dynamics have been studied using indirect measures such as fluorophotometry, tonography, venomanometry and anterior chamber depth [56–58]. Direct qualitative and quantitative analyses of diurnal AO have not previously been possible. Diurnal variation of IOP and aqueous outflow provides a challenge for HVI quantification techniques. Isolated measures of aqueous veins using HVI are likely to only represent a portion of true AO. Repeated HVI studies within a 24-h period and on subsequent days may help understand diurnal changes of AO and to correlate the findings with diurnal IOP changes.

### Effects of medications

Potential confounding effects of IOP-lowering eye drops on aqueous outflow patterns need to be considered. HVI studies have so far been observational and medication wash-out periods have not been possible. Cessation of IOP-lowering medications immediately after iStent *Inject* insertion led to unpredictable AO responses and 13% of eyes developed an IOP spike (>30% from baseline) 1 week after surgery [16]. The majority of eyes maintained post-operative IOP control, indicating factors other than drop cessation are likely to be contributing to IOP spikes. The use of topical anti-inflammatory drops, localised inflammation or attempts of the eye to regain IOP homoeostasis are other possible contributors [15, 16].

Topical drop therapies that promote aqueous outflow are commercially available in some countries [59, 60], but not yet in Australia. Specifically, no patient in any HVI study was taking a Rho-kinase inhibitor or latanoprostene bunod. Rho-kinase inhibitors are linked with high rates of conjunctival hyperaemia, which occurs due to blood vessel dilation from smooth muscle relaxation [59]. As a consequence, episcleral venous pressure may reduce, permitting additional aqueous drainage via the trabecular pathway [61, 62]. Using HVI to examine the effects of aqueous outflow-promoting medications (as seen with TBS) may clarify the therapeutic mechanism and contribute to our understanding of aqueous outflow regulation.

### Aqueous outflow measurement

HVI enables visualisation of AO within the episcleral venous system and the same vessel can be identified in successive scans for longitudinal comparison. The current quantification method using AqCA as a surrogate measure of sectoral AO is a simplified technique to demonstrate the effects of an intervention. AqCA does not provide an accurate representation of total AO volume or flow, so its use is not appropriate to quantify differences between eyes at baseline. Aqueous veins differ in size and length within each individual eye. Standardising the location of AqCA measurement is not possible due to infinite anatomical variations. Calculating the AqCA of every aqueous vein within an eye, then using an average, may provide a more accurate measure of AqCA and a closer representation of total AO. However, the number of aqueous veins varies greatly between subjects and some non-glaucomatous eyes have few visible aqueous veins. A more appropriate next step may be to calculate aqueous flow velocity and volume within an aqueous vein.

## Conclusion

Despite glaucoma being the most common cause of irreversible blindness in the world, comparatively little is understood of its pathophysiology. Treatment therefore empirically targets IOP reduction. Treatment selections are based on individual clinician opinion or preference rather than distinct clinical features of each patient. Greater knowledge of the aqueous outflow system is likely to contribute to individualised and precise glaucoma management. Despite its limitations, the use of HVI in multiple clinic-based studies has demonstrated the potential benefits of AO analysis and laid the foundations for future work.

HVI has confirmed AO findings that were first detected over 70 years ago, the importance of which were not fully appreciated until the recent introduction of MIGS. Redirection of aqueous drainage resulting from TBS is unpredictable, and will remain so, until a deeper understanding of glaucoma pathophysiology can be achieved. The justification of optimal stent placement within the nasal hemisphere, or even case selection based on aqueous outflow findings, is not yet possible. A collaborative approach combining detailed knowledge of each component of the aqueous outflow system, aided by emerging imaging technology, is required to expedite glaucoma diagnosis and optimise surgical outcomes.

## Data Availability

All reported data were derived from referenced publications.
